# When is digital documentation at its best? Swedish perioperative nurses’ experiences of digital documentation and its impact at their work environment: a qualitative study

**DOI:** 10.1136/bmjopen-2025-104968

**Published:** 2025-12-23

**Authors:** Björn Eriksson, Magnus Svartengren, Camilla Göras, Anna Dahlgren, Jessica Lindblom, Erebouni Arakelian

**Affiliations:** 1 Department of Medical Sciences, Occupational and Environmental Medicine, Uppsala University Uppsala, Uppsala, Sweden; 2 Department of Occupational and Environmental Medicine, Uppsala University Hospital, Uppsala, Sweden; 3 Faculty of Health and Occupational Sciences, Department of Caring Sciences, University of Gävle, Gävle, Sweden; 4 Department of Clinical Neuroscience, Karolinska Institutet, Stockholm, Sweden; 5 Human-Machine Interaction Unit, Department of Information Technology, Uppsala University, Uppsala, Sweden; 6 Department of Surgical Sciences, Uppsala University, Uppsala, Sweden

**Keywords:** Electronic Health Records, Nurses, QUALITATIVE RESEARCH

## Abstract

**Abstract:**

**Background:**

Digital documentation in patients’ electronic medical records (EMRs) places new demands on perioperative nurses, increasing workload and cognitive strain, with subsequent technostress. While new EMR systems are implemented, they are not always adapted to users’ needs.

**Aim:**

This study aimed to explore how perioperative nurses and nursing assistants describe their experiences with electronic documentation during surgery and its impact on their work environment. Additionally, it examined the emotional reactions these experiences triggered, and the adaptive strategies used to manage their effects.

**Methods:**

**Design:**

A qualitative study was conducted. Data were analysed using Braun and Clarke’s thematic analysis.

**Settings:**

Two university hospitals and one county hospital in Sweden were included.

**Participants:**

15 women and 5 men, including 9 specialist nurses in anaesthesia care, 9 nurses in operating room (OR) care and 2 nursing assistants in OR care in Sweden participated.

**Results:**

Two main themes emerged: A—Introducing digital systems without a clear aim undermines work performance and B—Digital systems were embraced when automation and comfort were present. Subthemes included leadership and management, possibilities to develop and influence digital systems and EMRs not adapted to clinical needs. Automation from digital systems made work easier, and digital systems within one’s comfort zone were appreciated. However, frustration and stress arose when aforementioned preconditions were not fulfilled, leading to adjustments to manage these challenges.

**Conclusions:**

Digital documentation is appreciated when fundamental conditions are met. A lack of clarity on how, what and why to document, along with insufficient training and limited ability to have an influence, triggers negative emotional reactions and unhealthy coping strategies. To enhance digital literacy, a standardised process of digital systems including digital documentation through educational efforts in which knowledge control in educational purposes is included could be tested as a potential solution.

**STRENGTHS AND LIMITATIONS OF THIS STUDY**

- Number of participants (n=20) might be considered a limitation; however, data were rich and no new information was found after 12 interviews.
- Interviews were conducted by phone and video due to participants’ preferences; no differences could be seen in the quality of these.
- Convenience sampling was used, which means that those who were interested in the topic (positively or negatively) participated.
- The gender imbalance may be considered as a limitation; however, it reflects the reality as the proportion of female nurses is higher in the field.
- Rich description of the context is a strength helping to decide transferability, which is done case-to-case.

## Introduction

 Documentation ensures the secure transfer of information between nurses and other healthcare professionals. The adoption of digital documentation in patients’ electronic medical records (EMRs) has increased, impacting both physical and cognitive aspects of the work environment.^1 2^ The digital work environment is a part of the overall work environment, encompassing organisational, social and physical factors shaped by digital support systems.^3^

EMRs store patients’ medical histories in digital health systems.^4^ Effective documentation requires digital literacy, including health informatics skills and knowledge of digital tools in patient care.^5^ In the perioperative context, covering care provided before, during and after surgery, specific EMR sections are used. The perioperative environment is dynamic and complex, often constrained by time and resources,^6^ with a strong emphasis on patient safety.^7^ Critical events, such as the induction or termination of anaesthesia, emergencies or haemorrhage, require immediate action from perioperative nurses, potentially disrupting the documentation process. Immediate patient care must proceed without reliance on EMRs, especially in such critical situations.^8^ Although perioperative nurses, such as registered nurse anaesthetists (RNAs) and operating room nurses (ORNs), work in the same operating room (OR), they are responsible for different documentation tasks across various EMR systems. There are other groups, such as surgeons or anaesthesiologists who also chart in patients’ EMRs. This study focuses on nurses’ and nursing assistants’ documentation.

### The physical work environment of digital documentation

Digital documentation creates new tasks,^9^ increasing both physical^10^ and cognitive workload, as one must process more information.^11 12^ Nurses have described dissatisfaction with the increased workload associated with patients’ EMRs, citing the excessive steps, variables and individuals involved.^13 14^ They reported spending 27% of their working hours on EMRs and 25% on direct patient care.^12^ Younger nurses (younger in age) particularly struggled with locating information and documenting patient care, leading to higher levels of stress and cognitive failures.^15^

### Cognitive ergonomics and technostress

Cognitive workload is defined as mental strain and effort required by working memory to complete tasks.^16^ Cognitive overload occurs when surgical demands and workflows are high^17^ or when excessive information must be processed.^18^ This situation necessitates that healthcare workers process a substantial amount of information, so-called cognitive ergonomics. The quantity of healthcare data has increased exponentially, exhibiting a 13-fold surge between 2013 and 2020.^19^ Therefore, it is crucial to effectively organise, simplify and mitigate potential risks to patient safety arising from increased physical and cognitive workloads.^20^

Technostress^21^ (or digital stress)^22^ is characterised by elevated stress levels resulting from the use of EMRs.^23^ Its origins lie in the organisational theory of sociotechnical systems, which comprises two fundamental components: the organisational and technological components. The former encompasses the social aspects, skills, attitudes and values, while the latter focuses on the work performed with the aid of technology. Technostress occurs when these two components are imbalanced.^21^ The load from technostress and a demanding work situation will not only affect decision-making and performance^24 25^ but can also lead to feelings of frustration, irritation and fatigue.^26 27^ In the short term, these reactions are important signals that performance may be affected^24 25^ and if these situations occur frequently over a longer period, they can signal a risk of burnout^28 29^ and leaving the occupation.^30 31^

Safe use of technology requires a multifaceted approach, including knowledge, education, training, organisational structure and leadership.^22^ Technostress can lead healthcare workers to develop workarounds–actions taken by professionals to compensate for deficiencies in technology.^32^ When flirting with safety margins without any incidents, clinical practice and shared values may drift towards the acceptance of unsafe behaviours and deviations from protocols, that is, normalisation of^24^ deviance.^33^ To minimise workarounds and technostress, it is essential to integrate the design of healthcare technology into the work system.^34^,^38^

### Human factors and ergonomics (HFE)

Neglecting HFE contributes to patient safety events, influenced by the usability of technology, system resilience, healthcare worker performance and human error.^39^ Neglecting HFE also affects the work environment, impacting staff well-being and job satisfaction.^39^ The systems engineering initiative for patient safety (SEIPS) model supports understanding and improving complex interactions within healthcare systems, aiming to enhance patient safety and quality of care. The SEIPS model assists in elucidating outcomes (system performance and human well-being) in complex sociotechnical systems. It identifies how aspects, such as tasks (eg, digital documentation) performed by humans (eg, perioperative nurses) using tools and technologies (eg, different EMRs) within a specific physical environment (here ORs) and organisation (eg, operating departments), affect outcomes.

The SEIPS model highlights the significance of clearly defining tasks, specifying required skills and providing education to ensure safe and efficient use of technology.^39^ It is essential for individuals, technology and organisations to cooperate and allow for sufficient preconditions (information, manpower, time and support) to effectively identify risks and make informed decisions. This collaboration will help reduce the physical demands of tasks, especially in already heavy workloads.^2^ To maintain consistent performance, it is vital to minimise cognitive load, facilitate error detection and learning from mistakes, enable continuous skill development, provide control over work systems and actively engage users in system design.^39^ The SEIPS model illustrates these interdependencies, showing how a lack of collaboration can compromise patient safety.

Since incidents often stem from the design of technologies, processes, workflows and sociotechnical systems,^40 41^ usability and safety in health Information technology (IT) are crucial.^39^ Cognitive requirements, such as attention, awareness and perception, along with social or behavioural requirements, including motivation and decision-making, and physical preconditions, that is, having sufficient time to perform a task, are essential.^39^

In summary, digital documentation in perioperative settings is essential for safe care. The implementation of the latest digital systems in EMRs in perioperative settings can increase cognitive workload, eventually causing technostress affecting decision-making, performance and emotional strain for the nursing staff and reducing time for direct patient care. Nurses often develop workarounds to adapt their workload to work requirements. While HFE frameworks, such as SEIPS, emphasise the need to align technology, people and organisation, limited knowledge exists about how perioperative nurses experience digital documentation and how they adapt to it.

## Aim

The aim was to explore how RNAs, ORNs and nursing assistants experienced electronic documentation during surgery and its impact on their work environment.

## Methods

### Design

This qualitative study was based on individuals’ experiences and thoughts,^42^ and followed the Consolidated Criteria for Reporting Qualitative Research checklist^43^ (Refer to online supplemental file 2).

### Settings and participants

Permission was obtained from the Heads of Departments participating in the study. Participants were given information about the study design, methods and how the results could be used in the clinical settings. Each individual was asked to participate via their work email, in accordance with ethical rules and regulations and informed consent was collected.

51 persons (ie, 18 RNAs, 24 ORNs and 9 nursing assistants) were asked to participate, of whom 20 participants accepted (of whom 5 were males). They were between 31 and 72 years of age (mean 48 years) and had between 2 and 53 years of work experience (mean 17 years). Of the RNAs (n=9) and ORNs (n=9), there were two males and seven females in each group, respectively. Of the nursing assistants (n=2), one was male. The participants were selected through convenience sampling. The participants were recruited from five different OR departments in two university hospitals and one county hospital in Sweden. The inclusion criteria comprised perioperative nurses and nursing assistants with experience in digital documentation. Administrative positions were excluded from the study. The results will be shared with the participating hospitals and with the research community.

### Data collection

The interview guide was designed by authors with experience in perioperative context, in occupational research and in human–computer interaction research. A semistructured interview guide with open-ended questions (refer to the interview guide in the online supplemental file 3) focused on how documentation was practised within patients’ EMRs, associated challenges and strategies to address them. Follow-up and probing questions were used to enhance the depth of the interview. The first interview, considered a pilot interview, was included in the analysis, as no changes were made to the guide afterwards.

Individual interviews were performed by author EA between April and August 2023, who has experience in qualitative research and the perioperative context. The interviews were between 35 and 69 min (mean 51 min) long and were conducted via video conference (n=3) or telephone (n=17). Only audio recordings were made. The interviews were transcribed verbatim for further analysis.

### Documentation in patients’ EMR in perioperative settings

In the OR, documentation is carried out by a team, including surgeons, an anaesthesiologist (who leaves after initiating anaesthesia), an RNA, an ORN and a nursing assistant. The nursing assistant supports the ORN in the sterile zone (and cannot touch unsterile materials, such as computers). The OR is divided into sterile and non-sterile zones, requiring clear collaboration and communication. The RNAs and ORNs have specific responsibilities. The RNAs record vital signs, blood loss and airway management on a digital or paper-based graph. The ORNs complete checklists and free text entries, focusing on data regarding the surgical procedure, including the instruments used and the number of swabs used.

There were no standardised documentation routines among the participants. One hospital used three different systems: one for OR scheduling, one EMR system for anaesthesia documentation and one main EMR for general medical records. Another hospital used a single EMR, with anaesthesia documentation recorded on paper, while the third hospital had two distinct EMRs: one for general documentation and another for anaesthesia. There were differences in the software used as EMR, and in the locations where information was documented. A common feature was that the different EMRs required separate logins, necessitating users to switch between them depending on the information needed at any given moment (at two hospitals).

### Data analysis

Data were analysed inductively using Braun and Clarke’s^44^ six-step thematic method (table 1). The findings resulted from discussions among all authors. No new information emerged after conducting 12 interviews; however, the remaining interviews were analysed to reduce the risk of overlooking important information.

**Table 1:** The thematic analysis steps according to Braun and Clarke

The thematic analysis steps according to Braun and Clarke	Authors who performed the step
Familiarisation phase: the interviews were read through several times to grasp the whole, and reflective notes were recorded.	BE, EA*
Coding and recoding: this process was carried out separately, followed by a reflection and discussion together.	BE, EA*
Based on similarities and differences in the codes, preliminary themes were identified.	BE, EA*
Themes were discussed with coauthors and revised accordingly.	BE and EA consulted CG, MS, JL and AD
The final themes were defined, and their content was described. With the themes in mind, the interviews were read through once more to ensure that they reflected the entire dataset.	BE, EA
The results were written and verified by the coauthors, who reviewed a selection of interviews to ensure that the themes emerged directly from the data.	BE and EA consulted CG, MS, JL and AD

EA, CG, AD and JL are female authors, and BE and MS are male authors. EA, CG and BE have experience from the perioperative context. EA, CG and MS have research experience in work environment. JL has research experience in human–computer interaction. CG and AD have research experience in patient safety research field. All authors had experience from qualitative research.

* Separately first and then together.

#### Methodological considerations

To verify the credibility and consistency^45^ of the results with the raw data, the authors used research triangulation and peer debriefing during the analysis process. Confirmability was achieved by presenting a detailed description of the analysis process, and quotations ensured that the findings were derived from the collected data. The authors also had experience in qualitative research, and in relevant areas, that is, occupational health, perioperative care and patient safety.

The issue of preunderstanding was discussed. Professional preunderstanding helps to understand the context and the interviews; however, there is a potential risk of overlooking familiar details. To minimise this risk, the text was read through multiple times.

The data were rich, meaning that the findings indicated that no new information was found after 12 interviews, indicating that the number of participants was sufficient. In qualitative research, the emphasis is placed on the ‘richness of data’ rather than the sheer number of participants, which was used to determine the sample size, confirming that it was adequate.^46 47^

In qualitative research, the transferability of the results is assessed on a case-by-case basis; thus, the results of the present study may be valuable in similar settings.

### Patient and public involvement

The patients and public were not involved.

## Results

Two themes and six subthemes were identified. Accompanying each theme are interrelating emotional reactions and adaptive strategies designed to mitigate these reactions (table 2), along with relevant quotations (table 3).

**Table 2:** An overview of the themes, subthemes, emotional reactions and adaptive strategies

Organisational preconditions and human–machine interactions	Emotional reactions	Adaptive strategies
**Theme A: introducing digital systems without a clear aim undermines work performance** Subtheme A1: perceived inadequate leadership and management Subtheme A2: unsatisfactory implementation process Subtheme A3: inability to adapt the digital systems Subtheme A4: EMR systems not designed for clinical needs	Frustration and fear Stress A sense of being let down	Using paper and manually writing down important information (overview) Finding their own way to document and help each other Avoiding important tasks, such as reading information about the patients in the EMRs
**Theme B: digital systems were embraced when automation and comfort were present** Subtheme B1: automation of work processes has positive benefits Subtheme B2: digital systems within one’s comfort zone are appreciated	Satisfaction	The adjustments have already been made

The emotional reactions of frustration, stress and a sense of being let down, along with subsequent adaptive strategies, arose due to theme A and its subsequent subthemes. In contrast, theme B and its associated subthemes did not lead to any adjustments, as employees were satisfied.

EMRs, electronic medical records.

**Table 3:** Themes and subthemes, quotations and emotional reactions

Themes and subthemes	Quotations	Emotional reactions and quotations
Theme A: introducing digital systems without a clear aim undermines work performance		
Subtheme A1: perceived inadequate leadership and management	*‘It’s (the documentation) all over the place–how people chart, both where things are documented and what is documented. Many people don’t know how to fill out some things, so they end up writing an entire essay someplace else, in a view where you can write free text on A, B, C, D and so on. So, it’s all over the place’. (NA 1*) *‘I don’t think it’s my job…we document statistics, but what does it mean for us. And above all, what are we going to do with it? It feels unnecessary. It seldom leads to anything (that benefits the employees). Does it mean we are going to hire more people?’ (NA 3*)	**Frustration** *‘One needs to dig deep into the system to find what is needed, and I don’t have time for that. Then, perhaps reading up on the patient might become a non-priority—you miss information, and then the patient safety is put in jeopardy’. (ORN 2*) *‘You can document antibiotics in three different places, which becomes dangerous if you forget to document in any of those places’*. (*NA 6*) *‘It’s a way for me to show that I’ve thought about these things, that I’ve checked it, to document this slide or this dose given, and so on. So if something goes wrong later, one can go back and check “what actually happened?”’ (ORN 9*) *‘(…)not everyone wants it that way, we want change… But then he (IT manager) can get really angry. He says it can’t be changed, and it’s really frustrating.(…)The administrators are going to reject at least 90 per cent of my suggestions’. (NA 6*) **Stress** *‘Once you’re done and exhausted (after a surgical case), you still have to complete documentation. Meanwhile, the person coordinating the schedule is already starting to talk about the next case, so yeah, that definitely causes stress’. (ORN 9*) *‘The surgical procedure takes 5–10 min, but the documentation afterwards takes 10–15 min… An angio…we do the procedure in 1 hour…and the documentation takes another 20–30 min… (ORN 1*) **A sense of being let down** ‘*When(…)was implemented, I was one of those who said “but we’ve been fooled!” They (the management))said we wouldn’t need to write something in that system, but in the end, they decided we had to do that anyway’. (NA 6*) *‘The documentation burden has become much more today…besides working with the operating site…documentation is more extensive today compared with the pre-digitalization era’. (ORN 5*)
Subtheme A2: unsatisfactory implementation process	*‘Education and introduction to the different systems were very scarce; there was no centralised education package. It was simply oral instructions from colleagues.’ (ORN 5*) *‘It must be difficult for someone who has never documented manually…we are not prepared for this (the internet had stopped working, thus the EMRs were out of use)’. (ORN 6*) *‘The EMR systems were out of use…the paper records were brought out. People felt insecure; none of us had worked with paper earlier. It’s not easy to know what to document…there has been no training for this scenario’. (NA 8*) *‘I don’t know if there is an action plan for this other than<go and get the paper records>…’. (NA 3*)	
Subtheme A3: inability to adapt the digital systems	*‘I brought this up in a meeting once because the change made things worse than it was before, but they (IT management) were like “no, we’re not going to change it back”, but obviously it was better before, but they made it worse. They don’t understand this, and they can get really angry’. (NA 6*) ‘*They created a “robot” to help transfer data, but humans are supposed to monitor the robot to make sure values don’t get stuck and that everything looks okay. Some units have staff who set aside part of their work hours to go through about 120 charts to make sure the data has been correctly transferred’. (NA 8*) *‘To be able to influence and develop the systems so that they are as safe as possible for the patient. In the end, that’s why we work here, to take care of the patient in the best possible way*’. (ORN 1).	
Subtheme A4: EMR systems not adapted for clinical needs	*‘One system tells me about the person, the human (the patient*)*, while the other system tells me about the procedure itself from a technical point of view, like what equipment I’ll need, what procedure is to be done, what kind of operating table do we need, and so on… So, I need them both…’*. (ORN 2) *‘For example, when you need to know the patient’s weight, but that information doesn’t transfer from one system to the other, so I need to go to the preoperative evaluation in another system to find it. But no, then the doctor hasn’t checked the weight, so then I need to go look in the third system, where you usually have to do like 3000 klicks to find the information you need’. (NA 9*)	

Theme B: digital systems were embraced when automation and comfort were present		
Subtheme B1: automation of work processes has positive benefits	*‘It’s quite satisfying not having to document the blood pressure manually’. (NA 6*) *‘Our scanner is like the ones at the grocery store; we scan a barcode on the bag of one of these disposable articles, for example. Not everything is registered, so some things we still need to enter manually, but quite a lot of articles are. Then we go to the ‘materials’ tab and just blip blip blip… Yeah, it really makes it easier’. ORN 3*	Positive feelings and satisfaction were expressed when systems were adapted to meet clinical needs. However, no further adaptations were made.
Subtheme B2: digital systems within one’s comfort zone are appreciated	“*I think they should just scrap a lot of systems… At least(the OR-scheduling system)is unnecessary for those of us working with anaesthesia. And typing in the ID number into the vital signs monitor… That could probably be done automatically’. NA 6*	

EMR, electronic medical record; NA, nurse anaesthetist; OR, operating room; ORN, operating room nurse.

## Theme A: introducing digital systems without a clear aim undermines work performance

To manage working with patients’ EMRs and digital documentation, several elementary prerequisites were described, such as leadership and management, involvement of the employees in the implementations process and possibility to influence the EMR systems, and EMRs adapting to employees’ clinical needs, which not always were to employee satisfaction. Several emotional responses, such as frustration and fear, stress and a feeling of being let down, arose in connection to different subthemes and adaptive strategies to handle these, which were described.

### Subtheme A1: perceived inadequate leadership and management

The lack of decision-making by management regarding documentation practices was highlighted by comments, such as *‘Nobody decides how to do it, and that leads to no one knowing how’* (RNA 6) and ‘*who has the mandate to decide*’ (RNA 8). Furthermore, a *‘lack of routines*’ (RNA 9) was noted, resulting in employees making their own interpretations about where and how to document certain information. There was uncertainty about the purpose of documenting or how it was ‘*beneficial for the future care of the patient; no one on the floor reads it’* (ORN 7). Concerns were also raised about data that are not immediately necessary for patient care, such as the statistics and timelines that were documented. Participants expressed the need for ‘*a unified system’* to avoid ‘*having 17 different things to document’* (ORN 9).

### Subtheme A2: unsatisfactory implementation process

No evaluations were performed after the implementation of the new digital systems. Participants reported that they received training at their workplaces when the new digital systems were implemented; however, continuing education and orientation for new employees relied on the competence of their colleagues or their individual interests.

RAN 4 stated that *‘one can learn documentation incorrectly if the preceptor doesn’t know how to document*’. As there were differences in knowledge of the EMRs, it was desirable to receive *‘more knowledge about the tool (EMRs) so that everyone can use it the same way’* (RNA 1). Participants also reflected on their own age and stated that it took longer for those who considered themselves ‘older’ to learn how to navigate separate EMRs.

Although examples, such as incorrect operating sites, surgeons failing to book necessary instruments and ordering antibiotics, were mentioned, no incident reports could be recalled by the participants regarding these issues. However, it is important to note that no patients were harmed due to routine controls carried out by ORNs and RNAs. In case the digital systems stopped working, the participants expressed uncertainty about the procedures for handling such a stoppage, particularly regarding the documentation of care, which would need to be completed through alternative means (eg, pen and paper). Some participants indicated that they would need to seek assistance from more senior colleagues, as they had never documented on paper before.

### Subtheme A3: inability to adapt the digital systems

The participants had limited influence over how to work and use digital systems, except for minor changes, such as moving a specific button or adding a drug specific to their unit. They also described that changes implemented by central management were not well integrated among the staff.

Changes were implemented to address various issues, sometimes making the digital systems difficult to navigate. However, typically, these changes only occurred when an external entity (such as the union or the Swedish Work Environment Authority) demanded that the issue be addressed. Occasionally, ‘home-developed’ solutions were created to serve as workarounds for the actual problem.

### Subtheme A4: EMR systems not adapted to clinical needs

Working with multiple EMR systems that do not share data can lead to information being scattered across these different systems. This meant that they became dependent on all systems, resulting in an increased workload to obtain the necessary information. RNA 6 explained that it required eight clicks across different systems to sign off and confirm that antibiotics had been administered.

Due to fragmentation across different EMRs, participants had to put in the same data multiple times, sometimes up to three times, resulting in an increased workload. One hospital had developed a ‘robot’ to transfer data from one system to another; however, due to several errors, a person was employed to monitor the robot/the machine.

### Emotional reactions

#### Frustration and fear

Participants described feeling frustrated when they were unsure whether they had all relevant or correct information. Sometimes, they had to call the surgeon to verify missing information or to confirm the details in the EMR. Frustration and concern for patient safety arose when documentation was forgotten in one of the systems due to scattered and duplicate record keeping. Another source of frustration was the neglect and dismissal of opinions and feedback by the digital system managers.

Participants expressed concerns about excessive documentation requirements and questioned the necessity of documenting data that seemed to be irrelevant for future care. The documentation was performed primarily due to liability concerns and a fear of being questioned later in case of incidents, particularly if the documentation was not done meticulously.

#### Stress

Stress was described due to time pressure, cognitive load and goal conflicts, for example, as not having time to document because of patient emergencies and the challenge of trying to remember what to document once things calmed down. This led to an increased cognitive workload, which involved engaging with the patient, noting the time of important events, and *‘documenting from one’s memory …which requires not forgetting the details…you must have a good memory…’* (RNA 5).

Excessive documentation was required between cases, as management rushed to start the next case. Participants also reported a conflict of goals and experienced stress from having to divide their attention between direct patient care and the documentation tasks. They felt compelled to dedicate more time to patient care; however, they were unable to do so because documentation required equal focus and time.

#### A sense of being let down

Participants described feeling deceived by management when new digital systems were implemented. The mere amount of data that needed to be documented and the subsequent workload increased significantly and gradually over time. They felt misled by management, who had created the impression that everything would be improved, but the reality turned out to be quite different.

### Adaptive strategies

To obtain the much-needed overview from the EMRs, participants consulted various systems and recorded the information on paper before starting each case or when reporting the patient between professions, such as RNAs and anaesthesiologists, or to the recovery unit through an oral report. RNA 4 explained that *‘80% of all RNAs in some capacity tear a piece of paper before each case and document on it…’*.

Participants also described that most staff had developed their own methods for documenting in the EMRs. Some documented specific information in one place, while others recorded it somewhere else, or in both places. They regularly taught and mentored each other. Allowing a colleague to handle the actual documentation was also a strategy used, particularly in acute situations during anaesthesia. A more regular strategy was for nursing assistants to help ORNs, which requires a high level of trust.

A third strategy was to bypass reading the medical records of the patient at hand during the preparation phase due to various reasons, such as a feeling of time shortage, which meant that vital information could be lost.

It was reported that *‘unfortunately, some older colleagues, don't read the records…it’s scary…’* (ORN8) before starting a case, as they simply did not think they had the time.

## Theme B: digital systems were embraced when automation and comfort were present

This theme described automation of work processes in working with EMRs and being comfortable with working with digital systems within one’s comfort zone, which led to satisfactory emotional responses.

### Subtheme B1: automation of work processes has positive benefits

The automation of digital systems was appreciated, as it eased the workload and time required for documentation. One notable example was the automated transfer of vital parameters from the monitoring systems to the EMR. One of the participating hospitals had used a solution for single sign-on, meaning that the user only had to sign in once with their work ID card to gain access to all systems. Participants from other hospitals also referred to this solution as something they had heard about and desired. Another example was the implementation of systems that enabled OR staff to scan barcodes of surgical materials and instruments, transferring that information to the EMR automatically.

### Subtheme B2: digital systems within one’s comfort zone are appreciated

Participants generally expressed a positive attitude towards the systems mainly used within their own professions, but they had a more negative attitude towards the system used by other professions. ORNs nurses were mostly positive about the OR-scheduling system, while RNAs appreciated the EMR used for documenting anaesthetic care. However, none of the participants appreciated the documentation in the EMR systems used on the wards, although they reluctantly complied with the requirement to document in the other systems than their own when instructed. They acknowledged that drawing strict boundaries regarding which profession uses which EMR might lead to an even more accentuated border between the different professional groups.

### Satisfaction

When systems were adapted for clinical use, users reported positive feelings and satisfaction.

## Discussion

Two main themes emerged, one describing elementary preconditions that were not fulfilled, which caused frustration and stress, and another theme about when digitalisation served its purpose and facilitated work. Subthemes included leadership and management, possibilities to develop and influence digital systems and EMRs not adapted to clinical needs. Automation from digital systems made work easier, and digital systems within one’s comfort zone were appreciated. Adjustments were made to manage these challenges.

We discuss our findings through the lens of the SEIPS model^39^ and emphasise the importance of preconditions, such as *organisation and management, the humans* performing a task, *the tool* used and the *information* provided, to ensure patient safety, in ‘*human–machine interaction’*. The model was used only after the inductive analysis as an interpretive framework in the discussion section.

The first theme of our findings pertains to the insufficient *organisation and management* of documentation tasks, that is, knowing about ‘what’, ‘where’ and ‘why’ different parameters in patient care should be documented, as requested by the informants in this study. Effective leadership, together with well-defined goals, is a necessary precondition to lead the way according to the SEIPS model to reach the goal of patient safety, which is also connected to workers’ job satisfaction.^39^

Another precondition in the SEIPS model^39^ was that *humans* using certain *technologies should* be able to learn from, influence and develop those technologies. To facilitate cooperation between *humans and technology*, the latter must support work processes rather than feel like a burden. In our study, a human was tasked with compensating for the shortcomings of the technology by monitoring and helping the ‘machine’; this dynamic should be the other way around, that is, the machine should support the human.

Some changes in personnel work processes may be required when new systems are used in an organisation. Therefore, some adaptations must be made,^40 41^ and designing trustworthy and credible with high usability technologies for safe patient care is essential.^39^ Adapting to these changes means integrating the new work tasks generated by digital systems^9^ with the existing ones. In contrast to the SEIPS model,^39^ the lack of *digital systems corresponding to the users’ needs* resulted in workarounds in our study. One workaround used was documenting from working memory, which resulted in cognitive overload.^16 17^ Workarounds may also reduce comparability in work processes and research across different hospitals. In an organisation, the employees need to be educated to guarantee digital literacy tailored to each employee’s needs. Our results indicated that older employees sometimes faced difficulties in learning new digital systems, contrary to the findings of Kaihlanen *et al*,^15^ who described that younger nurses encountered challenges in finding and documenting information related to patient care within these systems. We suggest implementing a standardised process of digital documentation through educational efforts in which knowledge control in educational purposes is included to ensure that the organisation achieves the necessary level of digital literacy.^48^ Moreover, barriers should be established to prevent the risk of ‘normal accidents’ occurring in ORs, for example, based on earlier incident reports.

According to the SEIPS model,^39^ accurate *transmission of information* is imperative for ensuring patient safety. This is achieved by facilitating interactions between the components, thereby establishing optimal preconditions for providing safe care. The lack of necessary preconditions, according to the SEIPS model,^39^ as identified in this study, led to *humans* developing negative feelings and emotional reactions, such as stress and frustration. In perioperative care, where patients’ conditions can change quickly, documentation was experienced as a competing demand, leading to increased technostress. Technostress^21^,^23^ may result in deviations from established practices, finding one’s own solutions or the normalisation of ‘new behaviours’, that is, normalisation of deviance,^33^ which may also jeopardise patient safety. Therefore, ensuring a safe organisational structure, simplifying processes and preventing potential risks associated with EMR usage are important for maintaining patient safety.^20^

Technostress has also been described as a lack of meaning in one’s work, which is fundamental for job satisfaction.^31^ The participants acknowledged uncertainties regarding how the documentation would be used later by other professions. This situation, where the work appears meaningless, led to some nurses neglecting to review patient information before starting a case, thereby shifting that responsibility to other team members. These nurses probably focused on the procedure due to information overload, information fatigue, stress or lack of time. Such neglect could jeopardise patient safety when vital information is potentially overlooked.

Documenting what was perceived as non-essential data input, such as process data or timelines, led to stress and a negative attitude towards digital documentation systems in general. These systems were viewed as increasing workload rather than making work more efficient. Consequently, documentation was seen as irrelevant to one’s specific area of care and, therefore, unnecessary. Employees must understand which data are necessary for a patient’s total care, including information needed for the next healthcare facility, the postoperative ward or the surgical ward, and ensure that it is documented in *one* place. When faced with information overload and scattered documentation, there is a risk that critical *information may be overlooked or lost*, which may threaten patient safety.

Perhaps, a salient observation is the duality of feelings towards digital documentation. In the second theme, digital documentation was described in positive terms, as it facilitated work when it served its intended purpose. Examples of effective human–machine interactions included the automated transfer of information from monitors to EMRs and the scanning of barcodes on surgical instruments. Importantly, in our study, the informants did not mention any unexpected events or ‘surprises’ caused by automation, which, if present, could indicate deficiencies in automation processes.

Interestingly, digital documentation has been used in Swedish healthcare for more than two decades, and many ‘essential problems’ should already have been resolved. However, it is crucial to address the need for *clear leadership and prerequisites for implementation and development of digital documentation in the organisation*, as well as the continuous involvement of different users, such as RNAs, ORNs and nursing assistants in the development of digital systems, particularly as there will be an introduction of new systems in the future. There also appears to be a need for a standardised approach to how EMRs are designed. This standard must align with the general care process to ensure that all nurses possess a basic skill level. Hence, small adjustments can be made according to the specific needs of each unit.

### Strengths and limitations

One limitation of the study is that it is focused on RNAs, ORNs and nursing assistants. The results show that digital documentation in perioperative care, described by the aforementioned categories, still incurred challenges despite digitalisation being a part of nursing care for more than two decades. The purpose of including different categories of nurses is to point to the complexity of the documentation in the patients’ EMRs and the EMR systems in use. The challenge cannot solely be addressed by one action and for one category, but it is a start. It requires a further comprehensive approach and looking at documentation from the different users’ views, such as nurses, surgeons and anesthesiologists.

The gender imbalance may be considered a limitation; however, it reflects the reality as the proportion of female nurses is higher in the field.

Interviews were conducted by phone and video due to participants’ preferences, but no differences could be seen in the quality of these.

Convenience sampling was used, which means that those who were interested in the topic (positively or negatively) participated. We aimed for a purposive sampling, but after receiving many declines in participation, we changed our strategy towards purposive sampling. The reasons for declining may be many; however, one reason might be the limitation to release nurses to participate in studies as they are stuck in the OR.

## Conclusion

Digital documentation is valued when elementary conditions are met, and the system supports the work processes for the employees effectively. However, a lack of knowledge regarding how, what and why to document, along with inadequate prerequisites, such as education and training and limited user influence, can lead to negative emotional reactions and adaptive strategies. Introducing a standardised process of documentation by educational efforts in which knowledge control in educational purposes is included could present a potential solution to achieve goals with good digital literacy, as well as a clear leadership, user involvement and clarification of purpose and goal of digital documentation.

## Supplementary material

10.1136/bmjopen-2025-104968 online supplemental file 1

10.1136/bmjopen-2025-104968 online supplemental file 2

10.1136/bmjopen-2025-104968 online supplemental file 3

## Data Availability

All data relevant to the study are included in the article or uploaded as supplementary information.
