# Supplementary material for: When is digital documentation at its best? Swedish perioperative nurses’ experiences of digital documentation and its impact at their work environment: a qualitative study
Source: BMJ Open. 2025 Dec 23;15(12):e104968. doi: 10.1136/bmjopen-2025-104968 (PMC12730763; doi:10.1136/bmjopen-2025-104968)
Supplement: online supplemental file 1 [file bmjopen-15-12-s001.docx]

**Supplemental file 1. Key Messages**

**What is already known on this topic**

The design of digital systems benefits from structure and a deep understanding of the user perspective.

**What this study adds**

Insights into the advantages and drawbacks of digitalisation from perioperative nurses’ perspectives, along with their proposals for improvement.

**How this study might affect research, practice or policy**

Research on digital systems can facilitate structured data collection if the user perspective is considered, supported by extensive education/ clear communication and well-defined objectives. These elements are essential for patient safety and a positive work environment.
