# Supplementary material for: When is digital documentation at its best? Swedish perioperative nurses’ experiences of digital documentation and its impact at their work environment: a qualitative study
Source: BMJ Open. 2025 Dec 23;15(12):e104968. doi: 10.1136/bmjopen-2025-104968 (PMC12730763; doi:10.1136/bmjopen-2025-104968)
Supplement: online supplemental file 2 [file bmjopen-15-12-s002.docx]

Supplemental File 2: COREQ checklist

Consolidated criteria for reporting qualitative studies (COREQ): 32-item checklist

Developed from:

Tong A, Sainsbury P, Craig J. Consolidated criteria for reporting qualitative research (COREQ): a 32-item checklist for interviews and focus groups. International Journal for Quality in Health Care. 2007. Volume 19, Number 6: pp. 349 – 357

| **Item No** | **Guide Questions/Description** | **Reported on Page #** |  |  |
| --- | --- | --- | --- | --- |
| **Domain 1: Research team and reflexivity** | | |  |  |
| **Personal Characteristics** | | |  |  |
| 1. Interviewer/ facilitator | Which author/s conducted the interview or focus group? | Pg 4 |  |  |
| 2. Credentials | What were the researcher’s credentials? E.g., PhD, MD | Title page |  |  |
| 3. Occupation | What was their occupation at the time of the study? | Title page |  |  |
| 4. Gender | Was the researcher male or female? | Table 3 |  |  |
| 5. Experience and training | What experience or training did the researcher have? | Pg 12, Table 3 |  |  |
| **Relationship with participants** | | |  |  |
| 6. Relationship established | Was a relationship established prior to study commencement? | Some were previous colleagues with EA and BE |  |  |
| 7. Participant knowledge of the interviewer | What did the participants know about the researcher? e.g. personal goals, reasons for doing the research? | Some were previous colleagues with EA and BE |  |  |
| 8. Interviewer characteristics | What characteristics were reported about the interviewer/facilitator? e.g. Bias, assumptions, reasons and interests in the research topic | Pg 12, methodological considerations |  |  |
| **Domain 2: study design** | | |  |  |
| **Theoretical framework** | | |  |  |
| 9. Methodological orientation and Theory | What methodological orientation was stated to underpin the study? e.g. grounded theory, discourse analysis, ethnography, phenomenology, content analysis | Pg 5, the abstract and table 3 |  |  |
| **Participant selection** | | |  |  |
| 10. Sampling | How were participants selected? e.g., purposive, convenience, consecutive, snowball | Pg 4 |  |  |
| 11. Method of approach | How were participants approached? e.g., face-to-face, telephone, mail, email | Pg 4 |  |  |
| 12. Sample size | How many participants were in the study? | Pg 4 |  |  |
| 13. Non-participation Setting | How many people refused to participate or dropped out? Reasons? | None |  |  |
| 14. Setting of data collection | Where was the data collected? e.g., home, clinic, workplace | On pg 4 there is information about the interviews being conducted by phone or video recordings.  The participants were either at their workplace or at home. The latter was not described in the paper. |  |  |
| 15. Presence of nonparticipants | Was anyone else present besides the participants and researchers? | No |  |  |
| 16. Description of sample | What are the important characteristics of the sample? e.g. demographic data, date | Table 1 |  |  |
| **Data collection** | | |  |  |
| 17. Interview guide | Were questions, prompts, and guides provided by the authors? Was it pilot tested? | Pg 4 |  |  |
| 18. Repeat interviews | Were repeat interviews carried out? If yes, how many? | No |  |  |
| 19. Audio/visual recording | Did the research use audio or visual recording to collect the data? | Pg 4 |  |  |
| 20. Field notes | Were field notes made during and/or after the interview or focus group? | Table 3 |  |  |
| 21. Duration | What was the duration of the interviews or focus group? | Pg 5 |  |  |
| 22. Data saturation | Was data saturation discussed? | Pg 5, Data analysis |  |  |
| 23. Transcripts returned | Were transcripts returned to participants for comment and/or correction? | N/A |  |  |
| **Domain 3: analysis and findings** | | |  |  |
| **Data analysis** | | |  |  |
| 24. Number of data coders | How many data coders coded the data? | Table 3 |  |  |
| 25. Description of the coding tree | Did the authors provide a description of the coding tree? | Figure 1, an example of the analysis process, was deleted due to limited number of figures and tables. However, it can be provided upon request |  |  |
| 26. Derivation of themes | Were themes identified in advance or derived from the data? | Pg 5, Results |  |  |
| 27. Software | What software, if applicable, was used to manage the data? | None |  |  |
| 28. Participant checking | Did participants provide feedback on the findings? | No |  |  |
| **Reporting** | | |  |  |
| 29. Quotations presented | Were participant quotations presented to illustrate the themes/findings? Was each quotation identified? e.g., participant number | Table 5, pg 5-9 |  |  |
| 30. Data and findings consistent | Was there consistency between the data presented and the findings? | Pg 5-9 |  |  |
| 31. Clarity of major themes | Were major themes clearly presented in the findings? | Table 4, Table 5, pg 5-9 |  |  |
| 32. Clarity of minor themes | Is there a description of diverse cases or a discussion of minor themes? | Pg 9-12, discussion |  |  |
