# Supplementary material for: When is digital documentation at its best? Swedish perioperative nurses’ experiences of digital documentation and its impact at their work environment: a qualitative study
Source: BMJ Open. 2025 Dec 23;15(12):e104968. doi: 10.1136/bmjopen-2025-104968 (PMC12730763; doi:10.1136/bmjopen-2025-104968)
Supplement: online supplemental file 3 [file bmjopen-15-12-s003.docx]

| Supplemental file 3. The interview guide | |
| --- | --- |
| Background question | Age, gender, occupation, experience, academic education |
| Main questions  (1-7)  **.** Follow-up questions | 1. How many IT- systems do you use in your daily life to perform documentation in patients’ electronic health records?  - Please explain how you perform documentation in patients’ EMRs before, during, and after surgery. - Describe a common wok day; what do you do to gather the necessary information about the patient? - What do you document and where? - How much documentation is remaining when the surgery is over/in the postoperative department?  1. What would you say are the challenges with digital documentation in your everyday work life?  - Please explain how and why? - How do you “solve” or “deal with” the challenges you have mentioned? - What are the benefits of the digital documentation system in patients’ EMRs?  1. How were you prepared for the digital documentation method in patients’ EMRs when you began working in your profession in the operating room?  - How did your workplace educate and prepare you for this task? - How were the new IT-systems introduced at your workplace? - Do you or did you have influence in adapting the documentation process to your needs? - How are evaluations conducted at your workplace to ensure that the IT- systems are designed to match your needs (after introducing new programmes)?  1. Do you know who made the decision to buy the IT-systems that are being used in your hospital?  - Do you know who makes the decision about what to document and where, and why? - Why do you document the way you do?  1. Let’s talk about patient safety issues: How do you discuss patient safety and other security aspects pertaining to digital documentation, particularly in connection to technical disturbances or hostile actions?  - In your opinion, how prepared are you if any hostile actions were to occur in your department? - How do you receive information about any deviations relating to digital documentation in patients’ electronic health/medical records?  1. Priorities and safety aspects regarding the patient: how do you prioritize between emergencies events (e.g., haemorrhaging or other situations requiring full focus on the patient) and documentation? 2. Is there anything you’d like to add? |
| Probing questions | Please tell more.  What do you mean? Please explain. |
