# Supplementary material for: When is digital documentation at its best? Swedish perioperative nurses’ experiences of digital documentation and its impact at their work environment: a qualitative study
Source: BMJ Open. 2025 Dec 23;15(12):e104968. doi: 10.1136/bmjopen-2025-104968 (PMC12730763; doi:10.1136/bmjopen-2025-104968)
Supplement: Supplementary file 4 [file bmjopen-2025-104968.reviewer_comments.pdf]

PEER REVIEW HISTORY

BMJ Open publishes all reviews undertaken for accepted manuscripts. Reviewers are asked to complete a checklist review form and are provided with free text boxes to elaborate on their assessment. These free text comments are reproduced below.

ARTICLE DETAILS

Title (Provisional)

When is digital documentation at its best? Swedish perioperative nurses' experiences of digital documentation and its impact at their work environment- a qualitative study

Authors

Eriksson, Björn; Svartengren, Magnus; Göras, Camilla; Dahlgren, Anna; Lindblom, Jessica; Arakelian, Erebouni

---

VERSION 1 - REVIEW

**Reviewer**	**1**
**Name**	**Lee, David**
**Affiliation**	**New York University Grossman School of Medicine**
**Date**	**15-Sep-2025**
**COI**	**None**

This study aimed to explore how perioperative nurses and nursing assistants described their experiences with electronic documentation during surgery.

Abstract: The themes that emerged are not intelligible as written. "elementary organizational preconditions" meaning what and what was found about them? "when digitalization served its purpose and facilitated work" when did it? Some of this is made more clear in the conclusions, but the results section should also speak for itself.

Introduction:

Line 57 on page 2: minor point but "has gradually increased" might be a severe understatement. Saying that it has increased should be sufficient.

It is interesting how the study decided to define and describe major terms to be used like cognitive workload, technostress, and identify human factors and ergonomics. While a bit unconventional, it appears to work in the context of this study.

Results: Theme A deserves some description of what exactly that means prior to moving onto sub-theme A1.

Line 50 on page 10 might need more context as it's not clear what this "journal" is and why it is scary that older colleagues don't read it.

Similarly, Theme B deserves some description of what it exactly means prior to the description of sub-theme B1.

Is there a reason why emotional responses and adaptive strategies are not really reflected on for Theme B subthemes?

Conclusions: The idea of certification comes out of the blue here in the conclusions and what does that mean? Doesn't adding yet another step that these nursing and nursing assistants have to do in getting certification add to the burden of their jobs? Is that a natural conclusion of the study and one even suggested by any of the interviewees? Was it asked of the participants whether that would even be a good idea?

Limitations of the study are not addressed.

Tables: The usefulness of table 1 is questionable and could maybe be replaced by text in the results communicating the same. Table 2 could also go as a supplemental table, in addition to Table 3. Given the presence of Table 5, Table 4 seems a bit unnecessary and some of its elements could be combined into Table 5.

**Reviewer**	**2**
**Name**	**Mukaihata, Tsuyoshi**
**Affiliation**	**Hyogo Medical University, Department of Nursing School**
**Date**	**16-Sep-2025**
**COI**	**None**

Thank you for the opportunity to review this manuscript. Please find my comments, particularly regarding the Methods section, outlined below.

1. Aims: While the Introduction provides a clear rationale for examining perceptions and impacts of electronic documentation, the justification for including emotional reactions and strategies as separate aims appears less fully developed. Expanding on the theoretical or empirical reasons for focusing on these aspects would strengthen the overall rationale for the study objectives.

2. Methods: The manuscript notes that RNs, ORNs, and nursing assistants are responsible for different documentation tasks across various EMR systems, and that there were no standardized documentation routines among the participants. While the inclusion of participants with diverse professional backgrounds may enrich the breadth and depth of the findings, it could also introduce variability that complicates interpretation. Could the authors elaborate on how they accounted for or reflected upon this diversity when analyzing and

discussing the results? A clearer consideration of this issue would likely strengthen the study’s validity and credibility.

3. Methods: I recommend relocating the section on “Methodological considerations” from the Discussion to the Data Analysis section. This adjustment would improve the coherence of the manuscript by ensuring that all aspects of the analytical procedure are presented consistently within the methods-related sections.

4. Methods: You have highlighted the importance of the SEIPS model in the Introduction and framed the discussion of the results in relation to this model. To enhance analytical transparency, it would be helpful if the authors could clarify whether the SEIPS model was used solely as an interpretive framework after the inductive analysis was completed, or if it shaped any part of the coding and analytical process. Providing such clarification would strengthen the methodological rigor of the study.

5. Discussion: I recommend that the authors create a distinct subsection entitled “Methodological limitations.”

**Reviewer**	**3**
**Name**	**Jung , James J**
**Affiliation**	**Duke University, Surgery**
**Date**	**22-Oct-2025**
**COI**	**None**

This qualitative study explores how perioperative nurses and nursing assistants experience electronic documentation during surgery, focusing on its impact on their work environment, emotional responses, and coping strategies. The study used semi-structured interviews and thematic analysis guided by the SEIPS model. This study provides timely evidence on the end-user's experience regarding use of electronic documentation.

I have a few minor revision comments:

1. Expand on the limitation. I think the discussion about the limitation should include sampling constraints (convenience sample, gender imbalance), data collection modes (phone vs video), and the eventual sample size. Emphasize transferability to similar high-resource perioperative settings.

2. Clarify the role of the SEIPS framework: explicitly describe how SEIPS informed the analysis. Discuss how it guided coding categories, interpretation, or theme development. A visual diagram showing how findings map onto SEIPS domains may enhance conceptual clarity.

**Reviewer**	**4**
**Name**	**Crowe, Francesca**
**Affiliation**	**University of Birmingham, Institute of Applied Health Research**
**Date**	**29-Oct-2025**
**COI**	**None**

When is digital documentation at its best? Perioperative nurses' experiences of digital documentation and its impact at their work environment- a qualitative study.

Review BMJ Open

The author s have conducted a really interesting study that raises a lot of important issues and questions in the field of health informatics and patient safety that are relevant globally. The manuscript is generally well written but some sections are light on detail and could be improved by describing some of the methods used in more detail.

Abstract, page 2

"Participants

Fifteen women and five men, i.e. nine specialist nurses in anesthesia.."

It might be better to phrase this as:

"Participants

Fifteen women and five men, including nine specialist nurses in anesthesia…"

It is not clear what "essential preconditions" are. Some clarification here and in the conclusion where this term is also used would be helpful.

Introduction

Page 3:

"In the perioperative context, covering care provided before, during, and after surgery, specific EMR sections are utilised. Although perioperative nurses, such as Registered Nurse Anesthetises (RNAs) and operating room nurses (ORNs), work in the same operating room (OR), they are responsible for different documentation tasks across various EMR systems."

Obviously there are other staff that are included in the operating room, so it might be helpful to include that perioperative nurses are responsible for completing the majority of EMRs in this setting (if this is the case) to help set the scene for the focus on nurses here?

“Younger nurses particularly struggled with locating…”

Is this younger nurses or early career nurses? Please clarify.

Page 5,

“Thus, digital documentation in perioperative settings is essential for safe care. The implementation of new digital systems in EMRs in perioperative settings can increase cognitive workload eventually causing technostress. Nurses often develop workarounds to adapt their workload to work requirements. However, studies exploring the specific impact of electronic documentation across perioperative settings remain limited.”

I think this paragraph could use a little more work to clearly develop and reference the ideas presented here leading to the rationale of the current study.

Methods

Setting and participant, page 5

How was the sample size decided upon?

Data collection, page 6

Could the authors add some detail on how the content for the interview guide was developed?

Were the audio recordings transcribed? Some more detail on how the authors went from the recordings to the data analysis would be helpful.

The Patient and Public Involvement, page 6.

Here, it is generally expected that you would detail how patients or the public were involved in this research. This could be that they were involved in helping to develop the research questions, they helped in developing the interview questions, or they may be involved in helping to communicate the findings. Sometimes, patients or the public are not involved in research so you just state that they were not involved.

I would suggest moving the information you have added under this heading to the heading “Setting and participants”. It would also be helpful to provide more details on how potential participants were approached, what information was provided to them about the study and how many were approached but decided not to participate.

Results

Sub-theme A2: Unsatisfactory implementation process, page 8

“Participants also reflected on their own age and stated that it took longer for them to learn how to navigate separate EMRs” – is this younger or older age or earlier/later career?

Discussion, page 11

It might help the reader to begin the discussion with a brief summary of the main findings before going on to discuss the findings through the lens of the SEIPS (Systems Engineering Initiative for Patient Safety) model?

Page 12

"The data was rich and became repetitive after 12 interviews..."

These two statements appear to be somewhat contradictory.

Table 1, page 19

Could you please define the abbreviations used here.

VERSION 1 - AUTHOR RESPONSE

Reviewer<br><br>1	Reviewer #1: Abstract: The themes that emerged are not intelligible as written. "elementary organizational preconditions" meaning what and what was found about them? "when digitalization served its purpose and facilitated work" when did it? Some of this is made more clear in the conclusions, but the results section should also speak for itself.	Thank you for your comment. The title of the themes has been revised. We hope now the themes headings describe their content more clearly. A- Digital systems with an unclear vision and B- Digital systems were embraced when automation and comfort were present. (Page 1)
	Introduction:<br>Line 57 on page 2: minor point but "has gradually increased" might be a severe understatement. Saying that it has increased should be sufficient.	The phrase has been revised to "has increased". Page 1.
	It is interesting how the study decided to define and describe major terms to be used like cognitive workload, technostress, and identify human factors and ergonomics. While a bit unconventional, it appears to work in the context of this study.	Thank you for your comment.

	Results: Theme A deserves some description of what exactly that means prior to moving onto sub-theme A1.	Thank you for your comment. We have added a description of theme A under the heading as follows: To manage working with patients' EMRs and digital documentation, several elementary prerequisites were described, such as leadership and management, involvement of the employees in the implementations process, and possibility to influence the EMR-systems, and EMRs adapting to employees' clinical needs, which not always were to employee satisfaction. Several emotional responses such as frustration and fear, stress and a feeling of being let down arose in connection to different subthemes and adaptive strategies to handle these which were described. Page 7.
	Similarly, Theme B deserves some description of what it exactly means prior to the description of sub-theme B1.	We have added a description of theme B under the heading as follows: This theme described automation of work processes in working with EMRs and being comfortable with working with digital systems within one's comfort zone, which led to satisfactory emotional responses. Page 11.
	Line 50 on page 10 might need more context as it's not clear what this "journal" is and why it is scary that older colleagues don't read it.	Thank you for your observation. We agree that additional text was needed here. Therefore, we have added the following text just before the quote: A third strategy was to bypass reading the medical records of the patient at hand at the preparation phase due to various reasons such as a feeling of time shortage, which meant that vital information could be lost.
	Is there a reason why emotional responses and adaptive strategies are not really reflected on for Theme B subthemes?	Please see the last paragraph under "Adaptive strategies" on Page 10.

	Conclusions: The idea of certification comes out of the blue here in the conclusions and what does that mean? Doesn't adding yet another step that these nursing and nursing assistants have to do in getting certification add to the burden of their jobs? Is that a natural conclusion of the study and one even suggested by any of the interviewees? Was it asked of the participants whether that would even be a good idea?<br><br>Limitations of the study are not addressed.<br><br>Tables: The usefulness of table 1 is questionable and could maybe be replaced by text in the results communicating the same. Table 2 could also go as a supplemental table, in addition to Table 3. Given the presence of Table 5, Table 4 seems a bit unnecessary and some of its elements could be combined into Table 5	Thank you for your comment. As satisfactory emotions in theme B were not articulated as much as the negative ones in theme A, these were not described as thoroughly as in theme A. Theme B and its associated sub-themes did not lead to any adjustments, as employees were satisfied.<br><br>Thank you for your comment. The question about a certificate was not asked. We rephrase the concept of "certificate" to "a standardized process of digital documentation through educational efforts in which knowledge control in educational purposes is included". This way everyone looking for relevant information would find it quicker and documentation would be safer. The standardized process of documentation will enhance patient safety in care. Page 13 and 16.<br><br>After abstract and in discussion section before conclusion we have extended the Methodological considerations with strengths and limitations. Page 15.<br><br>Thank you for your comment. Tables were created to meet the requirements of the journal's word limits.<br><br>We agree with you and submit Table 2 as supplemental file (file 3) and remove Table 1 (the information is written in text under "Participants"). However, we would like to keep Table 3, 4 and 5, (now Table 1, 2, 3) as they cover the data analysis steps, an overview of the themes/subthemes and their content and subsequent quotes.

2	1. Aims: While the Introduction provides a clear rationale for examining perceptions and impacts of electronic documentation, the justification for including emotional reactions and strategies as separate aims appears less fully developed. Expanding on the theoretical or empirical reasons for focusing on these aspects would strengthen the overall rationale for the study objectives.<br><br>2. Methods: The manuscript notes that RNs, ORNs, and nursing assistants are responsible for different documentation tasks across various EMR systems, and that there were no standardized documentation routines among the participants. While the inclusion of participants with diverse professional backgrounds may enrich the breadth and depth of the findings, it could also introduce variability that complicates interpretation. Could the authors elaborate on how they accounted for or reflected upon this diversity when analyzing and discussing the results? A clearer consideration of this issue would likely strengthen the study's validity and credibility.	Thank you for your comment. 1- We have added the following paragraph with subsequent references in the background of the study on page 2, 2nd paragraph, raws 6-10: The load from technostress and a demanding work situation will not only affect decision making and performance[24,25] but can also lead to feelings of frustration, irritation and fatigue.[26,27] In the short term these reactions are important signals that performance may be affected[24,25] and if these situations occur frequently over a longer period, they can signal a risk of burnout[28,29] and leaving the occupation[30,31].<br><br>2- We have also adjusted the aim of the study to "experiences" which includes emotional reactions.<br><br>Thank you for your valuable observation. We have addressed this issue under the heading "Strengths and limitations" after Methodological considerations before conclusion. One limitation of the study is that it is focused on RNAs, ORs and nursing assistants. The results show that digital documentation in perioperative care, described by the aforementioned categories still incurred challenges despite digitalization being a part of nursing care for more than two decades. The purpose of including different categories of nurses is to point to the complexity of the documentation in the patients' EMRs and the EMR systems in use. The challenge cannot solely be addressed by one action and for one category, but it is a start. It requires a further comprehensive approach and looking at documentation from the different users' views, such as nurses, surgeons, and anesthesiologists. Page5.

	3. Methods: I recommend relocating the section on “Methodological considerations” from the Discussion to the Data Analysis section. This adjustment would improve the coherence of the manuscript by ensuring that all aspects of the analytical procedure are presented consistently within the methods-related sections.	Page 14: Interestingly, digital documentation has been used in Swedish healthcare for more than two decades, and many “essential problems” should already have been resolved. However, it’s crucial to address the need for ***clear leadership and prerequisites for implementation and development of digital documentation in the organisation***, as well as the continuous involvement of different users such as RNAs, ORs, and nursing assistants in the development of digital systems, particularly as there will be an introduction of new systems in the future. <br>Thank you for your suggestion. We would like to keep the “Methodological consideration” in the discussion section. However, we have added “Strengths and limitations” to Methodological considerations and extended the discussion about the study strengths and limitations. Page 15.
	4. Methods: You have highlighted the importance of the SEIPS model in the Introduction and framed the discussion of the results in relation to this model. To enhance analytical transparency, it would be helpful if the authors could clarify whether the SEIPS model was used solely as an interpretive framework after the inductive analysis was completed, or if it shaped any part of the coding and analytical process. Providing such clarification would strengthen the methodological rigor of the study.	Thank you for your comment. The SEIPS model was used solely as an interpretive framework after the inductive analysis. Information was added in the first paragraph of the discussion section: Page 11: We discuss our findings through the lens of the SEIPS model[28] and emphasise the importance of preconditions, such as ***organisation and management, the humans*** performing a task, ***the tool*** used, and the ***information*** provided, to ensure patient safety, in

	5. Discussion: I recommend that the authors create a distinct subsection entitled "Methodological limitations."	***"human-machine interaction"***. The model was used only after the inductive analysis as an interpretive framework in the discussion. (Page 12)<br><br>After abstract and in discussion section before conclusion we have added a section about strengths and limitation of the study to Methodological considerations. Page 15.
3	1.Expand on the limitation. I think the discussion about the limitation should include sampling constraints (convenience sample, gender imbalance), data collection modes (phone vs video), and the eventual sample size. Emphasize transferability to similar high-resource perioperative settings.<br><br>2. Clarify the role of the SEIPS framework: explicitly describe how SEIPS informed the analysis. Discuss how it guided coding categories, interpretation, or theme development. A visual diagram showing how findings map onto SEIPS domains may enhance conceptual clarity.	Thank you for your comment. After abstract and in discussion section before conclusion we have added a section about strengths and limitations of the study to Methodological considerations, where we have expanded the discussion on sample size and the issues of transferability. Page 15.<br><br>Thank you for your comment. The SEIPS model was used solely as an interpretive framework in the discussion section, after the inductive analysis in the discussion section. Information was added in the first paragraph of the discussion section:<br><br>Page 12: We discuss our findings through the lens of the SEIPS model[28] and emphasise the importance of preconditions, such as ***organisation and management, the humans*** performing a task, ***the tool*** used, and the ***information*** provided, to ensure patient safety, in ***"human-machine interaction"***. The model was used only after the inductive analysis as an

		interpretive framework in discussion section.
4	Abstract, page 2<br><br>"Participants<br>Fifteen women and five men, i.e. nine specialist nurses in anesthesia.."<br><br>It might be better to phrase this as:<br>"Participants<br>Fifteen women and five men, including nine specialist nurses in anesthesia…"<br><br>It is not clear what "essential preconditions" are. Some clarification here and in the conclusion where this term is also used would be helpful.	Thank you for your comment. We have rephrased as you have suggested.<br><br>Thank you for your comment. We have clarified it with adding the word "aforementioned" before preconditions as it refers to leadership and management, possibilities to develop and influence digital systems, and EMRs not adapted to clinical needs.<br><br>Results (in the abstract)<br><br>Two main themes emerged: A- Digital systems with an unclear vision and B- Digital systems were embraced when automation and comfort were present. Sub-themes included leadership and management, possibilities to develop and influence digital systems, and EMRs not adapted to clinical needs. Automation from digital systems made work easier, and digital systems within one's comfort zone were appreciated. However, frustration and stress arose when aforementioned preconditions were not fulfilled, leading to adjustments to manage these challenges. Page 1.

	Introduction<br>Page 3:<br>"In the perioperative context, covering care provided before, during, and after surgery, specific EMR sections are utilised. Although perioperative nurses, such as Registered Nurse Anesthetises (RNAs) and operating room nurses (ORNs), work in the same operating room (OR), they are responsible for different documentation tasks across various EMR systems."<br><br>Obviously there are other staff that are included in the operating room, so it might be helpful to include that perioperative nurses are responsible for completing the majority of EMRs in this setting (if this is the case) to help set the scene for the focus on nurses here?	Thank you for your comment. We have added a sentence to clarify the focus of the study being the nursing staff:<br><br>Page 2, in the end of the introduction section: In the perioperative context, covering care provided before, during, and after surgery, specific EMR sections are utilised. Although perioperative nurses, such as Registered Nurse Anesthetises (RNAs) and operating room nurses (ORNs), work in the same operating room (OR), they are responsible for different documentation tasks across various EMR systems. There are other groups such as surgeons or anaesthesiologists who also chart in patients' EMRs. This study focuses on nurses and nursing assistants' documentation.
	"Younger nurses particularly struggled with locating…"<br><br>Is this younger nurses or early career nurses? Please clarify.	Thank you for your observation. Younger refers to younger in age which was added to the following sentence on page 2:<br><br>Younger nurses (younger in age) particularly struggled with locating information and documenting patient care, leading to higher levels of stress and cognitive failures.[12]
	Page 5,<br>"Thus, digital documentation in perioperative settings is essential for safe care. The implementation of new digital systems in EMRs in perioperative settings can increase cognitive workload eventually causing technostress. Nurses often develop workarounds to adapt their workload to	Thank you for your comment. This paragraph is meant as a summary of rationales for the study. Therefore, there are no references in this paragraph. The references are in the background of the study. We have

	work requirements. However, studies exploring the specific impact of electronic documentation across perioperative settings remain limited."<br><br>I think this paragraph could use a little more work to clearly develop and reference the ideas presented here leading to the rationale of the current study.	made the following changes to mark this clearly.<br><br>1-We have removed the paragraph about perioperative environment to introduction section. Please see page 2, 2nd paragraph, rows 4-9.<br><br>2-We have re-written the summery/rationales as (Page 4): In summary, digital documentation in perioperative settings is essential for safe care. The implementation of new digital systems in EMRs in perioperative settings can increase cognitive workload eventually causing technostress; affecting decision making, performance and emotional strain for the nursing staff and reduce time for direct patient care. Nurses often develop workarounds to adapt their workload to work requirements. While Human Factors and Ergonomics (HFE) frameworks, such as SEIPS, emphasize the need to align technology, people, and organization, limited knowledge exists about how perioperative nurses experience digital documentation and how they adapt to it.
	Methods<br>Setting and participant, page 5<br>How was the sample size decided upon?	On page 15: According to both Malterud et al (2016) and Guest et al (2020) identification of "new information" or "data saturation" (the term used in Grounded theory, and not here) and richness of data, in combination with researcher's experience can be used to estimate the sample size. We used this "method" to decide the sample size. Qualitative studies often require between 20-30 interviews to reach the state richness of data which was met after 12 interviews as no new information was found thereafter. We

		have added text and the following reference in the Methodological considerations where sample size is discussed: "The data was rich, meaning that the findings no new information was found after 12 interviews, indicating that the number of participants was sufficient. In qualitative research, the emphasis is placed on the "richness of data" rather than the sheer number of participants, which was used to determine the sample size, confirming that it was adequate.[47,48]". Additional reference: Guest G, Namey, E. Chen M. A simple method to assess and report thematic saturation in qualitative research. PLoS One. 2020 May 5;15(5). doi: 10.1371/journal.pone.0232076.
	Data collection, page 6<br>Could the authors add some detail on how the content for the interview guide was developed?	Thank you for your comment. In the beginning of "Data collection" section we added the following sentence to clarify this:<br><br>Page 5: The interview guide was designed by the authors who have experience in perioperative context, in in occupational research, and in human-computer interaction. A semi-structured interview guide with open-ended questions (Please refer to the interview guide in the supplementary file) focused on how documentation was practised within patients' EMRs, associated challenges, and strategies to address them. Follow-up and probing questions were used to enhance the depth of the interview. The first interview, considered a pilot interview, was included in the analysis as no changes were made to the guide afterwards.
	Were the audio recordings transcribed? Some more detail on how the authors went from the recordings to the data analysis	Page 6: Thank you for pointing that out. We have added a sentence at the

	would be helpful.<br><br>The Patient and Public Involvement, page 6.<br><br>Here, it is generally expected that you would detail how patients or the public were involved in this research. This could be that they were involved in helping to develop the research questions, they helped in developing the interview questions, or they may be involved in helping to communicate the findings. Sometimes, patients or the public are not involved in research so you just state that they were not involved.<br><br>I would suggest moving the information you have added under this heading to the heading "Setting and participants". It would also be helpful to provide more details on how potential participants were approached, what information was provided to them about the study and how many were approached but decided not to participate.<br><br>Results<br>Sub-theme A2: Unsatisfactory	end of the "Data collection" section to clarify this: The interviews were transcribed verbatim for further analysis. The analysis steps were described in Table 2 on page 21.<br><br>Page 7: Thank you for your guidance. We have changed the text under the heading: The Patient and Public Involvement to: Patient and Public were not involved.<br><br>We have removed the information above to Setting and participants:<br><br>Page 5: Permission was obtaioned from the Head of Departments participating in the study. Information was given about the study design, methods and how the results could be used in the clinics. Each participant was asked to participate individually through the work e-mail, according to ethical rules and regulations and informed consent was gathered. The results will be shared with the participating hospitals, and with the research community.<br><br>Page 5: Fifty-one persons (i.e. 18 RNAs, 24 ORNs, and nine nursing assistants) were asked to participate of whom 20 participants accepted (of whom five were males). They were between 31-72 years of age (mean 48 years) and had between 2-53 years of work experience (mean 17 years). Of the RNAs (n=9) and ORs (n=9) there were two males in each group. Of the nursing assistants (n=2), one was male. The participants were selected through convenience sampling.<br><br>Thank you for your observation. This refers to age as in how old the person

	implementation process, page 8<br><br>"Participants also reflected on their own age and stated that it took longer for them to learn how to navigate separate EMRs" – is this younger or older age or earlier/later career?	is. Meaning that when one gets older it, they notice difficulties with learning how to navigate in the EMRs. We have added a few words to clarify this: Participants also reflected on their own age and stated that it took longer for those who considered themselves "older" to learn how to navigate separate EMRs. Page 8.
	Discussion, page 11<br><br>It might help the reader to begin the discussion with a brief summary of the main findings before going on to discuss the findings through the lens of the SEIPS (Systems Engineering Initiative for Patient Safety) model?	Thank you for your comment. We had chosen not to write a summery and follow the word limits of the journal. We have now added a summary of the results as requested (Page 12): Two main themes emerged, one describing elementary preconditions that were not fulfilled which caused frustration and stress, and another theme about when digitalization served its purpose and facilitated work. Sub-themes included leadership and management, possibilities to develop and influence digital systems, and EMRs not adapted to clinical needs. Automation from digital systems made work easier, and digital systems within one's comfort zone were appreciated. Adjustments were made to manage these challenges.
	Page 12<br><br>"The data was rich and became repetitive after 12 interviews…"<br><br>These two statements appear to be somewhat contradictory.<br><br>Table 1, page 19	Thank you for your observation. We revised the sentence as follows and we hope that it is clearer now (Page 15): The data was rich, meaning that the findings no new information was found after 12 interviews…

	Could you please define the abbreviations used here.<br><br>Reviewer: 1<br>If you have selected ‘Yes’ above, please provide details of any competing interests.: Not applicable.<br><br>Reviewer: 2<br>If you have selected ‘Yes’ above, please provide details of any competing interests.: Not applicable<br><br>Reviewer: 3<br>If you have selected ‘Yes’ above, please provide details of any competing interests.: Not applicable<br><br>Reviewer: 4<br>If you have selected ‘Yes’ above, please provide details of any competing interests.: Not applicable	Thank you for your comment. Table 1 was removed as requested by another reviewer.

VERSION 2 - REVIEW

**Reviewer**	**1**
**Name**	**Lee, David**
**Affiliation**	**New York University Grossman School of Medicine**
**Date**	**18-Nov-2025**
**COI**	

The study is much improved. However, I would recommend that authors still describe what Theme A means "Digital systems with an unclear vision" - did what? Digital systems with an unclear vision of ____ were [not well] received? Or something similar.

VERSION 2 - AUTHOR RESPONSE

Formatting Amendments (where applicable):	We cannot see in the document where formatting is required, but we are willing to do so if there are specific suggestions.
Dr. David Lee, New York University Grossman School of Medicine, New York University School of Medicine<br>Comments to the Author:<br>The study is much improved. However, I would recommend that authors still describe what Theme A means "Digital systems with an unclear vision" - did what? Digital systems with an unclear vision of ____ were [not well] received? Or something similar.	Dear Dr. David Lee, thank you for your comment. We have revised Theme A to A- Introducing digital systems without a clear aim undermines work performance.<br><br>We hope that the new heading of Theme A describes the content more clearly. However, we are willing to make further changes if required.
If you have selected 'Yes' above, please provide details of any competing interests.: Not applicable	I apologize for the mistake. There is no conflict of interest to report. I have now selected the correct choice, which is "No". The following text is disclosed in the box: The authors report no conflict of interest. This study was funded by the Swedish Research Council for Health, Working Life and Welfare, grant #2021-01561.
